# Serum markers of B‐cell activation in pregnancy during late gestation, delivery, and the postpartum period

**DOI:** 10.1111/aji.13090

**Published:** 2019-01-30

**Authors:** Jorge Lima, Geraldine Cambridge, Andreia Vilas‐Boas, Catarina Martins, Luís‐Miguel Borrego, Maria Leandro

**Affiliations:** ^1^ Department of Obstetrics and Gynecology CUF Descobertas Hospital Lisbon Portugal; ^2^ Department of Immunology, Chronic Diseases Research Center (CEDOC), Faculty of Medical Sciences NOVA Medical School Lisbon Portugal; ^3^ Centre for Rheumatology and Bloomsbury Rheumatology Unit, Division of Medicine University College London London UK; ^4^ Department of Immunoallergy CUF Descobertas Hospital Lisbon Portugal

**Keywords:** B lymphocytes, B‐cell‐activating factor, biomarkers, female, free light chains, immunoglobulins, pregnancy, soluble CD23

## Abstract

**Problem:**

B cells are vital for the normal evolution of pregnancy due to their humoral and possible regulatory activities. Our group and others have documented that circulating B‐cell subsets undergo changes from normal late pregnancy to the postpartum period. However, the underlying mechanisms are poorly understood. Therefore, this study examined the degree of B‐cell activation in normal pregnancy by analyzing the levels of serum markers in healthy pregnant women during the third trimester of pregnancy, the day of delivery, and the postpartum period.

**Method of study:**

A prospective study including pregnant and non‐pregnant women attending routine care was undertaken at a hospital clinic. Sociodemographic and clinical data were collected, along with peripheral blood samples. The serum levels of soluble CD23 (sCD23), B‐cell‐activating factor (BAFF), kappa (κ) and lambda (λ) free light chains (FLC), IgA, IgG, and IgM were quantified.

**Results:**

Our study included 43 third trimester pregnant and 35 non‐pregnant women. In the pregnant women, the median levels of sCD23, BAFF, IgG, and κ FLC were significantly higher during the postpartum period than during the third trimester of pregnancy. Compared to the non‐pregnant women, the third trimester pregnant women had higher median BAFF levels and lower sCD23, IgA, IgG, and FLC levels.

**Conclusion:**

Changes in serum markers of B‐cell kinetics that occur during pregnancy often persist into the postpartum period and affect the secretion of immunoglobulins from different classes. Further studies are needed to clarify the biological significance of our observations.

## 1 INTRODUCTION

Successful pregnancy requires major regulation of the maternal immune system without compromising its role in the protection of either the mother or fetus.^1^ B cells play an essential role in pregnancy because they are paramount for humoral activity and the production of antibodies, which are also implicated in the normal evolution of pregnancy. Nevertheless, B cells may cause obstetric complications, such as pregnancy loss, preeclampsia, intrauterine growth restriction, stillbirth, and preterm birth, through the production of autoantibodies.^2^, ^3^, ^4^

We have previously reported that peripheral blood B‐cell subsets undergo quantitative changes from the third trimester of pregnancy to the postpartum period in healthy pregnant women.^5^, ^6^, ^7^ According to our study, late pregnancy is accompanied by peripheral blood B‐cell lymphopenia followed by a recovery of B‐cell levels later during the postpartum period.^5^ Recent studies that have assessed gene expression in maternal plasma and in the placenta by RNA sequencing have further confirmed this hypothesis.^8^, ^9^ Moreover, pregnancy‐associated B‐cell lymphopenia has already been described in animal models^10^, ^11^ and humans^12^, ^13^, ^14^, ^15^, ^16^, ^17^, ^18^, ^19^, ^20^, ^21^, ^22^ and probably occurs due to hormonal influences^10^, ^11^ and/or cellular migratory mechanisms during the later stages of pregnancy.^23^, ^24^, ^25^, ^26^

Although the biological significance of the suppression of B‐cell lymphopoiesis in normal pregnancy is still unclear, this suppression may be related to the physiology of maternal‐fetal immune tolerance.^10^

Additional data addressing changes in B‐cell biology during pregnancy can be obtained by the analysis of serum markers of B‐cell function, such as via measurements of the levels of B‐cell‐activating factor (BAFF), serum soluble CD23 (sCD23), immunoglobulins (Ig), and free light chains (FLC), which are elevated in several autoimmune diseases, namely, rheumatoid arthritis,^27^, ^28^ Sjogren syndrome,^29^ and systemic lupus erythematosus (SLE).^30^ Additionally, elevated serum concentrations of BAFF have been described in B‐cell malignancies and in primary and acquired immunodeficiencies,^31^, ^32^, ^33^, ^34^ although whether these increases are correlated with qualitative or quantitative changes in the circulating B‐cell compartment remains unknown. Higher BAFF levels may be a physiological response to ensure the survival of transitional B cells and to support the expansion of a growing B‐cell compartment.^35^ In healthy adults, soluble BAFF levels are very low, suggesting that the amounts of BAFF produced are the quantities that are necessary to provide sufficient survival signals to maintain and limit the size of the steady‐state B‐cell pool.^36^ Strikingly, Muzio et al^11^ showed that the numbers of pre‐/pro‐ and immature B cells were progressively diminished during pregnancy in the bone marrow of pregnant mice, leading to a reduction in B‐cell influx into the blood and spleen. Correspondingly, lower BAFF levels were observed in the sera of pregnant mice. In addition, Bienertova‐Vasku et al^37^ recently demonstrated that the BAFF levels in circulating human maternal blood were significantly higher in women with normal pregnancies than in pregnant women with preeclampsia.

CD23 is expressed on earliest B cells exiting the bone marrow while the post‐germinal center and other memory B cells are negative for this marker.^38^ Following B‐cell activation, as the surface expression of CD27 induces cleavage of CD23, sCD23 levels can be used as a measurement of B‐cell turnover from naïve to memory B cells.^39^ The soluble factor sCD23 is stable for 12‐24 hours in serum and has been used by us and others as a surrogate measure of naïve B‐cell activation or differentiation.^28^ Excess FLC are released into the circulation by terminally differentiated B cells, antibody‐producing cells. The evaluation of serum FLC levels has been shown to be useful for monitoring the activity of autoimmune diseases such as lupus,^29^, ^30^ and serum FLC levels may even be superior to the usual biomarkers such as complement, double‐stranded DNA, and serum Ig.^30^

Little is known about whether markers of B‐cell activation and differentiation change during normal human pregnancy. Therefore, the aim of this study was to explore the relationship between the levels of serum markers of B‐cell activation and B‐cell maturation into memory and Ig‐secreting cells during late pregnancy and the postpartum period of healthy pregnant women. Specifically, this study uses quantitative data from enzyme‐linked immunosorbent assays (ELISAs) to determine the extents to which the levels of serum sCD23, BAFF, serum kappa (κ) and lambda (λ) FLC, IgA, IgG, and IgM change from the third trimester of pregnancy and the day of delivery to the postpartum period and compare these values to those of non‐pregnant women. This report presents one of the first investigations of the degree of B‐cell activation in normal pregnancy. This characterization is important for our increased understanding of the resumption of autoimmune responses and their associations with B‐cell kinetics following delivery and the postpartum period.

## 2 MATERIALS AND METHODS

### 2.1 Study design and participants

This is a prospective observational study conducted under the scope of our project on the characterization of the circulating B‐cell populations of healthy non‐pregnant and pregnant women from the third trimester of pregnancy to the postpartum period.^5^

All women were enrolled in the study between July 2013 and March 2014 at Hospital CUF Descobertas in Lisbon (Portugal). Consecutive nonlaboring pregnant women who were receiving routine prenatal care at a hospital outpatient clinic during the third trimester of pregnancy were eligible for this prospective study. Third trimester pregnant women were included in the study if they had no prior pregnancy complications and if their fetuses exhibited appropriate growth, as assessed by uterine fundal height and by an ultrasound performed after 28 weeks of gestation. Exclusion criteria for third trimester pregnant women were multiple pregnancy, ongoing pregnancy complications (including preeclampsia), delivery of a premature infant (born before 37 weeks of gestation), induced labor, signs of infection, or prenatal use of any medication other than vitamins and iron supplementation.

As a control group, we recruited consecutive healthy non‐pregnant women who were attending routine annual well‐woman examinations at the hospital outpatient clinic. Exclusion criteria for non‐pregnant women were the use of hormonal oral contraceptives, as these drugs may affect the levels of peripheral blood lymphocyte subsets.^40^ Furthermore, the following exclusion criteria were applied for both pregnant and non‐pregnant women: diabetes; hypertension; autoimmune diseases; vascular diseases; HIV, syphilis, or hepatitis B or C infection; or smoking within 6 months prior to study inclusion.

From each pregnant woman, we collected peripheral blood samples during three planned clinic visits: (a) during the third trimester of pregnancy; (b) on the day of delivery, within 15 minutes of placental expulsion and oxytocin administration; and (c) during the postpartum period, at least six weeks after delivery. From each non‐pregnant woman, we collected a single peripheral blood sample at one planned clinic visit during the follicular phase of her menstrual cycle because the luteal phase may change the levels of peripheral blood lymphocyte subsets in a manner that is similar to that of pregnancy.^41^

The present study quantified the serum levels of the following B‐cell markers: sCD23, which is enzymatically cleaved and released from naïve B cells following activation; BAFF (also known as TNFSF13B or BLyS protein); κ FLC and λ FLC, as measures of plasmablast activity; and IgA, IgG, and IgM.

All procedures performed in our study involving human participants were conducted in accordance with the ethical standards of the institutional research committee and with the 1964 Declaration of Helsinki and its later amendments or comparable ethical standards. This article does not contain any studies with animals performed by any of the authors. The hospital's Ethics Committee approved our study protocol, and all participants provided their signed informed consent prior to their inclusion in the study.

### 2.2 Baseline characteristics

Baseline data collected for all study participants included age, ancestry, body mass index, blood pressure, and obstetric history. Data collected for the pregnant women included gestational age at the first planned visit (third trimester of pregnancy) and on the day of delivery; usage of obstetrical anesthesia and/or analgesia; and mode of delivery. Data collected for the newborns included sex, birth weight, and 1‐ and 5‐minutes APGAR scores.

### 2.3 Sample collection and measurements

Peripheral blood samples were collected into tubes without any anticoagulant agent and were centrifuged after coagulum retraction. Serum was separated, aliquoted, and stored at −20°C until further analysis. The serum concentrations of human CD23/FcεRII (pg/mL) and human BAFF/BLyS/TNFSF13B (pg/mL) were quantified using the Quantikine® ELISA commercial kits (R&D Systems Europe Ltd, Abingdon, UK) according to the manufacturer's instructions. The mean minimum detectable doses of these kits were 3.18 pg/mL and 2.68 pg/mL for human CD23/FcεRII (pg/mL) and human BAFF/BLyS/TNFSF13B, respectively.

The total IgA, IgG, and IgM serum concentrations (g/L) were determined using in‐house nephelometry by Binding Site Group Ltd. (Birmingham, UK).

The human κ FLC and λ FLC serum concentrations (mg/L) were analyzed using Freelite, a commercial ELISA kit (Binding Site), according to the manufacturer's instructions. The limits of detection for these kits were 6 and 5 μg/L for human κ FLC and λ FLC, respectively. The κ/λ FLC ratio was also calculated and used as an indicator of clonal expansion.

### 2.4 Statistical analysis

The characteristics of the study participants and levels of the serum markers of immune activation were summarized using descriptive statistics. The normality of the data was assessed using the Shapiro‐Wilk test. Continuous variables are presented as the mean (standard deviation) or median (minimum‐maximum), as applicable. Two independent groups were assessed using Student's *t*‐test or the Wilcoxon rank‐sum test, as applicable. Paired data were compared using paired Student's *t*‐tests or the Wilcoxon signed‐rank test, as appropriate. Comparisons between more than two groups were performed using analysis of variance (ANOVA), the Kruskal‐Wallis test, or the Friedman test, as applicable. For the assessment of correlations, Spearman correlation coefficients were calculated. *P*‐values for all comparisons were adjusted for multiplicity using the Benjamini and Yekutieli method.^42^ Categorical samples were expressed as numbers and percentages and compared using Fisher's exact test. Statistical significance was concluded at the level of 0.05. This was an exploratory study in which no calculation was performed to determine the sample size. All data analyses were conducted with R Core Team, Vienna, Austria.

## 3 RESULTS

### 3.1 Baseline characteristics

During the eight‐month study period, 48 third trimester pregnant women were enrolled in the study. Five pregnant women were excluded from further analysis due to the delivery of a premature infant (n = 3), delivery outside the study site (n = 1), or technical difficulties (n = 1). Only the data of the remaining 43 third trimester pregnant women were included in our analysis. In addition, 35 non‐pregnant women were included in the study.

The baseline characteristics of the non‐pregnant women and of the third trimester pregnant women and their newborns are shown in Table 1. Briefly, the median age of the third trimester pregnant women was 32.0 years (25‐41), and 55.8% were nulliparous. Their mean body mass index was 26.2 (2.8) kg/m^2^, their mean systolic blood pressure was 115.7 (9.3) mm Hg, and their mean diastolic blood pressure was 67.4 (7.4) mm Hg. The median gestational ages were 33.0 (31‐35) weeks and 39.0 (37‐41) weeks in the third trimester of pregnancy and on the day of delivery, respectively. The mean infant birth weight for 43 newborns was 3265.0 (393.5) g. All pregnant women received obstetrical anesthesia and/or analgesia regardless of the mode of delivery, and no general anesthesia was administered. In general, the pregnant women were discharged from the hospital 2 days after a vaginal delivery or 3 days after a cesarean section. The planned clinic visits during the postpartum period occurred at a median time of 45 (range 41‐58) days after delivery. Regarding the non‐pregnant women, their median age was 35.0 (range 20‐40) years, and 40% were primiparous. Their mean body mass index was 21.5 (2.8) kg/m^2^, their mean systolic blood pressure was 119.8 (10.5) mm Hg, and their mean diastolic blood pressure was 74.7 (7.4) mm Hg. For the non‐pregnant women, the median time since their last pregnancy was 169 (range 23‐449) weeks (including live birth or an interrupted pregnancy).

**Table 1:** Baseline characteristics of non‐pregnant women and of third trimester pregnant women and their newborns

Characteristics	non‐pregnant (n = 35)	Pregnant (n = 43)
Age, median (minimum‐maximum), y	35.0 (20‐40)	32.0 (25‐41)*
Body mass index, mean (SD), kg/m^2^	21.5 (2.8)	26.2 (2.8)
Blood pressure, mean (SD), mm Hg		
Systolic	119.8 (10.5)	115.7 (9.3)
Diastolic	74.7 (7.4)	67.4 (7.4)
Ancestry, no. (%)		
European	35 (100)	42 (97.8)
African	0	1 (2.2)
Parity, no. (%)		
Nulliparous	5 (14.3)	24 (55.8)***
Primiparous	14 (40.0)	18 (41.9)
Multiparous	16 (45.7)	1 (2.3)
Gestational period, median (minimum‐maximum), wk		
Third trimester of pregnancy		33.0 (31‐35)
Day of delivery		39.0 (37‐41)
Mode of delivery, no. (%)		
Vaginal		18 (41.8)
Cesarean		25 (58.2)
Newborns		
Male, no. (%)		22 (51.0)
Birth weight, mean (SD), g		3265.0 (393.5)
APGAR score, median (minimum‐maximum)		
1‐min		9 (6‐10)
5‐min		10 (9‐10)

SD, standard deviation.

* *p*‐value <0.05;

*** *p*‐value <0.001.

The third trimester pregnant women were significantly younger (*P* = 0.016) and included significantly more nulliparous women (*P* < 0.001) than the non‐pregnant women. Nevertheless, no significant correlations were found between age and the levels of the studied B‐cell markers, namely, sCD23, BAFF, κ FLC, λ FLC, IgA, IgG, and IgM, for either the pregnant women or non‐pregnant women (data not shown). Similarly, no significant differences were found in the measured serum parameters between the different parities, within the group of third trimester pregnant women or within the group of non‐pregnant women (see Table S1), or between vaginal compared to cesarean delivery at third trimester, day of delivery, or postpartum period in the group of third trimester pregnant women (see Table S2).

### 3.2 BAFF levels are higher in third trimester pregnant women than in non‐pregnant women

**Figure 1:**
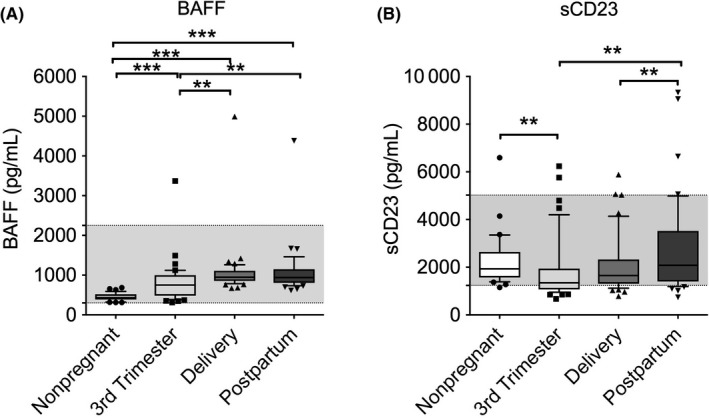
Box (median ± interquartile ranges) and whiskers (10th and 90th percentiles) of the serum BAFF (A) and sCD23 (B) levels in the peripheral blood of non‐pregnant women (n = 35) and pregnant women (n = 43) during the third trimester of pregnancy, on the day of delivery within 15 min after placental expulsion, and during the postpartum period at least 6 wk after delivery. The gray area indicates the range of normal values. Differences between groups were tested using analysis of variance (ANOVA) or the Friedman test. *P‐value <0.05; **P‐value <0.01; ***P‐value <0.001

We have found that the median BAFF levels were significantly increased in pregnant women at all study visits compared to non‐pregnant women (third trimester of pregnancy, *P* < 0.001; day of delivery, *P* < 0.001; postpartum period, *P* < 0.001; Figure 1A). In the third trimester pregnant women, BAFF levels were significantly higher on the day of delivery (*P* = 0.007) and during the postpartum period (*P* = 0.005) than during the third trimester of pregnancy.

We did not find significant correlations between the serum BAFF levels and the absolute or relative circulating B‐cell levels (see Table S3).

### 3.3 Soluble CD23 levels increase during the postpartum period

The median sCD23 serum levels varied throughout the third trimester of pregnancy to the postpartum period in the pregnant women and between the third trimester pregnant women and non‐pregnant women (Figure 1B). Specifically, the sCD23 levels were significantly lower in the pregnant women during the third trimester of pregnancy than in the non‐pregnant women (*P* = 0.002). Moreover, the sCD23 levels increased significantly in the pregnant women during the postpartum period compared to the third trimester of pregnancy (*P* = 0.004) and to the day of delivery (*P* = 0.005).

### 3.4 IgA levels are lower in third trimester pregnant women and decrease on the day of delivery

**Figure 2:**
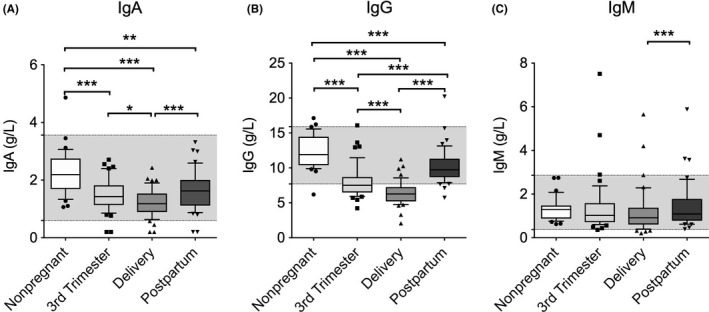
Box (median ± interquartile ranges) and whiskers (10th and 90th percentiles) of the total serum levels of (A) IgA, (B) IgG, and (C) IgM in the peripheral blood of non‐pregnant women (n = 35) and pregnant women (n = 43) during the third trimester of pregnancy, on the day of delivery within 15 min after placental expulsion, and during the postpartum period at least 6 wk after delivery. The gray area indicates the range of normal values. There was one missing value for IgA in the pregnant women group. Differences between groups were tested using analysis of variance (ANOVA) or the Friedman test. *P‐value <0.05; **P‐value <0.01; ***P‐value <0.001

The median IgA levels were significantly lower in the third trimester pregnant women, regardless of the timing of the clinic visit, than in the non‐pregnant women (third trimester of pregnancy, *P* < 0.001; day of delivery, *P* < 0.001; postpartum period, *P* = 0.009; Figure 2A). In addition, the median IgA levels decreased significantly on the day of delivery compared to the third trimester of pregnancy (*P* = 0.007) and increased significantly from the day of delivery to the postpartum period (*P* < 0.001).

### 3.5 IgG levels are lower in third trimester pregnant women and often below the normal range at delivery

The median IgG levels showed a similar tendency to that of the IgA levels, being significantly lower in the pregnant women during all visits than in the non‐pregnant women (Figure 2B; *P* < 0.001). In the third trimester pregnant women, the lowest median IgG levels were detected on the day of delivery and increased significantly during the postpartum period compared to both the day of delivery (*P* < 0.001) and the third trimester of pregnancy (*P* < 0.001). In contrast to the changes observed in the IgA levels, the IgG levels were significantly higher during the postpartum period than during the third trimester. Both during the third trimester and, particularly, at delivery, the IgG levels were often below the normal range.

### 3.6 IgM levels are not different between pregnant and non‐pregnant women

There were no significant differences in the median IgM levels between the pregnant and non‐pregnant women (Figure 2C). Moreover, in the third trimester pregnant women, the median IgM levels only showed a slight but significant increase during the postpartum period compared to the day of delivery (*P* < 0.001).

### 3.7 The κ/λ FLC ratio increases during the postpartum period

**Figure 3:**
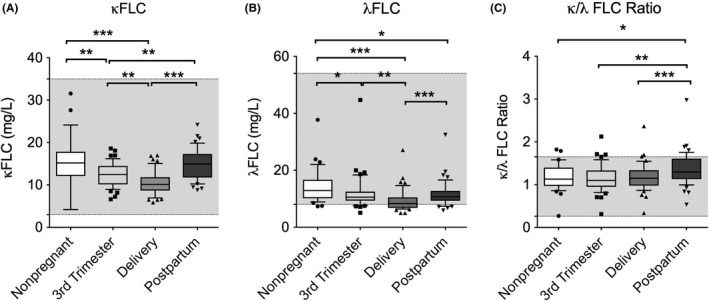
Box (median ± interquartile ranges) and whiskers (10th and 90th percentiles) of the (A) κ FLC levels, (B) λ FLC levels, (C) and κ/λ FLC ratio in the peripheral blood of non‐pregnant women (n = 35) and pregnant women (n = 43) during the third trimester of pregnancy, on the day of delivery within 15 min after placental expulsion, and during the postpartum period at least 6 wk after delivery. The gray area indicates the range of normal values. Differences between groups were tested using analysis of variance (ANOVA) or the Friedman test. *P‐value <0.05; **P‐value <0.01; ***P‐value <0.001

The serum FLC levels followed the same pattern as that of the measured class‐switched Ig (IgA and IgG) levels, with the median levels of both κ and λ FLC being significantly higher during the postpartum period than on the day of delivery (Figure 3A,B). The levels of κ FLC recovered during the postpartum period and were not significantly different from those of the non‐pregnant women, whereas the levels of λ FLC remained low. This was reflected by the changes in the κ/λ FLC ratio (Figure 3C).

## 4 DISCUSSION

In our previous study, we found that the levels of some peripheral blood B‐cell subsets significantly vary throughout the third trimester of pregnancy until the postpartum period.^5^ Thus, we hypothesized that these changes might be accompanied by variations in markers of immune activation, namely, sCD23 and BAFF, given their reported relationship with B‐cell differentiation and transitional B‐cell survival, respectively. However, the literature is scarce regarding the expression of markers of B‐cell activation during normal human pregnancy. Therefore, in the present study, we aimed to address this problem by quantifying the serum levels of sCD23, BAFF, κ and λ FLC, IgA, IgG, and IgM in the peripheral blood of healthy pregnant women (third trimester of pregnancy, the day of delivery, and postpartum period) and non‐pregnant women.

Soluble CD23 levels in the peripheral blood have been previously explored in human pregnancy, comparing women with normal gestations (only during the first trimester) to women who experienced spontaneous abortions, but there was no difference between the two groups.^43^ We found that the serum sCD23 levels were significantly lower in the pregnant women during the third trimester of pregnancy and increased during the postpartum period. Considering that the presence of sCD23 fragments in the circulation is thought to depend on the turnover from naïve to memory B cells, our findings are consistent with our previous report,^5^ as we identified a relative increase in naïve B cells in the third trimester of pregnancy (ie, at a time of lower sCD23 levels). Moreover, the rise in sCD23 levels during the postpartum period suggests the resumption of B‐cell differentiation into memory B cells, also supported by our previous results.^5^ Interestingly, in patients with rheumatoid arthritis undergoing rituximab therapy, we (ML and GC) have previously found that rises in sCD23 levels were highly associated with relapse and coincided with the increased production of IgM rheumatoid factor and serum FLC release.^28^ Elevated concentrations of these fragments have also been frequently associated with inflammatory or lymphoproliferative disorders.^44^, ^45^

Another finding was that the BAFF levels were significantly higher in the third trimester pregnant women than in the non‐pregnant women but remained largely within the normal limits. In addition, in the pregnant women, the BAFF levels were significantly higher on the day of delivery and during the postpartum period than during the third trimester of pregnancy. These results reflect those of Lundell et al,^46^ who found that the BAFF levels in maternal plasma were higher at 30 days after delivery than at 19 days before giving birth. Nevertheless, the mechanisms that regulate the circulating levels of BAFF are not well understood in humans. This outcome is contrary to that reported by Muzzio et al,^11^ who showed that BAFF levels were decreased in the sera of pregnant mice; however, this discrepancy may be due to the different dependencies of human and murine B cells on BAFF signaling.

Several factors can explain our observation. First, BAFF is essential for B‐cell survival and the differentiation of immature transitional B cells into mature naïve cells, and BAFF is an important immune regulator that has been reported to be secreted by the placenta.^47^, ^48^ A previous study demonstrated that BAFF exhibits higher expression in the trophoblasts and decidua of normal early pregnant women than in the tissues of recurrent spontaneous abortion patients,^49^ confirming that BAFF may be an essential element in the implantation of the embryo and maintenance of pregnancy. In addition, findings from a recent study have suggested that decidual stromal cells, stimulated with interferon (IFN)‐γ and IFN‐α, are a cellular source of BAFF for the B cells present in the decidua, and this can be of importance for local B‐cell homeostasis during pregnancy.^50^ Prior studies have observed an in vivo correlation between BAFF levels and B‐cell numbers in patients with rheumatoid arthritis receiving B‐cell depletion therapy or who have primary antibody deficiencies.^28^, ^36^ Following B‐cell depletion, progressively increasing levels of serum BAFF may signify an incremental decrease in the total B‐cell pool, as in the context of immunodeficiency, peripheral B‐cell numbers and the expression of BAFF receptors have been shown to be inversely proportional to the concentration of serum BAFF.^36^

We have previously reported that compared to non‐pregnant women, a normal healthy pregnancy between the third trimester and delivery is associated with a decreased circulating B‐cell compartment that is richer in naïve B cells and poorer in more differentiated subsets, such as memory cells and plasmablasts.^5^ The biological consequences of this B lymphopenia in normal pregnancy are uncertain but probably related to the physiology of immune tolerance. Nevertheless, in the present study, the serum BAFF levels, which are usually positively correlated with plasmablast generation,^51^ were increased in the healthy third trimester pregnant women. The serum BAFF levels in the group of non‐pregnant women evaluated here were similar to those previously reported in non‐pregnant non‐obese women in another study.^52^ Therefore, the cause‐effect relationship between BAFF levels and B‐cell subsets during pregnancy remains to be elucidated.

Interestingly, recent studies have shown that hormones such as estrogens can modulate the expression of BAFF.^53^, ^54^ Additionally, pregnancy hormones have also an impact in circulating B cells, which seems to suggest that the hormone milieu of pregnancy may induce B‐cell lymphopenia, thus the relative availability of BAFF receptors will also be limited, which may lead to an increase in serum BAFF levels. Therefore, increased levels of serum BAFF might be an effect of B lymphopenia in normal pregnancy. However, in the puerperium, B cells are known to recover their circulating levels, and still, we observed increased levels of BAFF, even when compared to the third trimester. This suggests other regulatory mechanisms in the regulation of this molecule. Eventually, BAFF receptors may suffer a modulation in the postpartum as observed in autoimmune conditions.^55^ Additionally, the hormonal milieu significantly changes after labor, and it may also interfere with the regulation of BAFF pathways. Nevertheless, our results showing increased BAFF levels in the maternal serum on the day of delivery and during the postpartum period can represent a strong B‐cell maturation boost for the mother giving birth, as part of a compensatory mechanism that acts on the B‐cell compartment to maximize the maternal‐fetal defense capacity. In this context, BAFF might contribute to the increase in and maturation of B cells in the mother for recovery after B‐cell lymphopenia, and this can also explain the higher serum levels of IgG and IgM that we observed during the postpartum period. However, caution is due here because increased levels of serum BAFF have been associated with an amplified B‐cell response with consequent autoimmunity^33^ and may eventually interfere with the course of pregnancy in immunocompromised women or in those with autoimmune diseases. BAFF levels in maternal peripheral blood have been explored in preeclamptic pregnancies in a few studies; however, the results have been variable, with some reports showing no changes^2^ and others showing a significant decrease^37^ in serum BAFF in women with preeclampsia comparing with normotensive pregnant women. Studies with larger sample sizes may be required to further investigate if BAFF can be a possible biomarker for preeclampsia.

The current study found that the κ/λ FLC ratio increases during the postpartum period, which can be related to a higher activity of the adaptive immune system. This finding is an indicator of B‐cell activation occurring after the systemic immunological reaction associated with childbirth. In addition, the κ/λ FLC ratio provides a sensitive numerical indicator of clonality.^56^ Thus, its increase suggests that polyclonal hypergammaglobulinemia occurs during the postpartum period and may be caused by an inflammatory state.

Regarding whether there is variation in the serum levels of Ig, we found that IgA levels were lower in the third trimester pregnant women than in the non‐pregnant women and decreased further on the day of delivery; the IgG levels were lower in the third trimester pregnant women at all times of assessment, failing to recover by the postpartum period. The IgM levels were not different between the pregnant and non‐pregnant women (showing a significant increase during the postpartum period compared to on the day of delivery). The major Ig in peripheral blood have been investigated in normal human pregnancy and during the postpartum period, but the results have been highly variable.^57^, ^58^, ^59^, ^60^ Recently, Ziegler et al^61^ addressed the immunoglobulin profile along pregnancy. Though our results are partially in line with this study, for IgM and even IgA, our results are mostly discrepant in what concerns IgG. We found an important decrease in IgG during the third trimester, but Ziegler et al,^61^ which evaluated IgG subclasses instead, showed that pregnancy interferes differentially with each IgG subclass and that IgG1 is always increased in pregnant women compared to non‐pregnant. This inconsistency can be attributed to differences in study design, as some studies did not include the postpartum period and not all of the women in other studies had samples for all time points. Other likely causes include differences in laboratory methodologies, sample size, gestational age, ethnicity, environments, and ecosystems. Second, although the levels of most Ig remain unchanged during pregnancy, reductions in the levels of IgA and IgG have been reported by other authors.^58^, ^59^, ^60^ These falls during pregnancy may be due to hemodilution, the leaking of IgG through urine, the transfer of IgG from mother to fetus across the placenta during the third trimester, or the pregnancy‐associated milieu of hormones, especially steroid hormones, which have effects on protein synthesis.^59^, ^62^ Moreover, the levels of both IgA and IgG were lower in the third trimester pregnant women, suggesting that IgM precursors may not predominate in circulation during late pregnancy or on the day of delivery. Finally, the increase in both serum Ig levels and the κ/λ FLC ratio during the postpartum period can be explained by an expansion of specific B cells into Ig‐secreting cells.

In general, therefore, it seems that a reinstatement of an anti‐inflammatory state occurs after delivery, suggesting that there is a qualitative immunological dynamic change in pregnant women that is closely associated with changes in the serum levels of sCD23, BAFF, FLC, and IgG. This study supports evidence from our previous report regarding the quantitative changes in the peripheral B‐cell compartment during the third trimester to the postpartum period.^5^ One of the issues that emerges from these findings is the need to carefully clarify the impact of pregnancy‐associated changes in the B‐cell compartment on humoral immune function, as the use of maternal immunization to protect the health of the pregnant woman, her fetus, and the infant has increased over the past decade. Policy changes for vaccines aimed at protecting both mothers and their infants during the first months of life have occurred, and many health authorities are now recommending immunizations during pregnancy.^63^, ^64^, ^65^ These new strategies have been reinforced by a recent study demonstrating that tetanus, diphtheria, and pertussis vaccination during pregnancy increases antibody titers against diphtheria and pertussis, which may prevent neonatal pertussis infection.^66^ Further immunological insights can be useful for the improvement of maternal vaccination programs to increase their safety and efficiency.

This study has several strengths to be highlighted. First, to obtain a homogenous sample and reduce potential bias, we only included women with normal singleton pregnancies who delivered between 37 and 41 weeks of gestation. Moreover, this was a prospective study, with all clinical monitoring (including deliveries) and laboratory tests performed by the same research team and under the same protocol. Second, all the women contributed samples at all time points, namely, during the third trimester, on the delivery day, and during the postpartum period. In addition, we also accounted for the phase of the menstrual cycle during which the samples were collected from the non‐pregnant women. However, due to its observational nature, our study has some limitations. We consecutively included all patients who were followed at the outpatient clinic and fulfilled the eligibility criteria for which no sample size calculation was carried out. However, even with the small sample size, our results clearly showed significant differences between the studied markers. Nevertheless, caution must be applied, as the findings might not be generalizable to the broader population. Additional prospective studies should be performed using larger sample sizes and samples from additional time points, such as during the first and second trimesters of pregnancy, and should monitor women prior to becoming pregnant through the postpartum period. Future research should also compare immune cell subsets in the peripheral blood and decidua at delivery from the same healthy pregnant women.

In conclusion, to our knowledge, this is the first study to report increases in several markers of B‐cell activation during pregnancy, namely, BAFF, and during the postpartum period, namely, sCD23 and the κ/λ FLC ratio. The most obvious finding to emerge from this study is that B‐cell activation is an immunological event in pregnancy that continues into the postpartum period and affects the secretion of Ig molecules from several classes. This study provides important insights into the role of B lymphocytes in the immunomodulation of normal pregnancy and may help explain the increased susceptibility to infection seen during pregnancy.^67^, ^68^ Another theoretical implication of this study is that the role of the placenta as an immunological organ may be important for further investigation in the context of autoimmune diseases such as SLE, as there are reports^69^, ^70^ showing an increased rate of SLE flares during pregnancy, and other adverse obstetric outcomes such as pregnancy loss, fetal growth restriction, preeclampsia, and preterm birth. Likewise, the occurrence of autoimmune diseases during the postpartum period is not uncommon.^71^, ^72^ Further prospective and functional studies are mandatory to clarify the importance of our observations.

## CONFLICT OF INTEREST

The authors have no conflict of interest to declare.

## AUTHOR CONTRIBUTIONS

Jorge Lima and Maria Leandro conceived the original research idea, while all the authors designed the study and created the study protocol. Jorge Lima recruited the patients and collected the data. Andreia Vilas‐Boas analyzed the blood samples. Maria Leandro and Luís‐Miguel Borrego supervised all the work and the research protocol. All the authors contributed to data analysis and interpretation. Jorge Lima drafted the manuscript, and all the authors revised it and contributed to it intellectually. All the authors have approved the final version of the manuscript.
