# Supplementary material for: Serum markers of B‐cell activation in pregnancy during late gestation, delivery, and the postpartum period
Source: Am J Reprod Immunol. 2019 Jan 30;81(3):e13090. doi: 10.1111/aji.13090 (PMC6590212; doi:10.1111/aji.13090)
Supplement: Supplementary file 1 [file AJI-81-na-s001.docx]

**Supplemental Tables**

**Supplemental Table S1.**

Levels of serum markers of B cell activation in different parities within the groups of nonpregnant women and pregnant women.

| **Serum marker** |  | **Nonpregnant women (n = 35)^†^** | | |  | **Pregnant women (n = 43)^†^** | | | | | | | | | | |
| --- | --- | --- | --- | --- | --- | --- | --- | --- | --- | --- | --- | --- | --- | --- | --- | --- |
|  |  |  |  |  |  | **3^rd^ trimester** | | |  | **Day of delivery** | | |  | **Postpartum period** | | |
|  |  | *Nulliparous* | *Primiparous* | *Multiparous* |  | *Nulliparous* | *Primiparous* | *Multiparous* |  | *Nulliparous* | *Primiparous* | *Multiparous* |  | *Nulliparous* | *Primiparous* | *Multiparous* |
| **BAFF, pg/mL** |  | 526.8 (121.1)^‡^ | 428.5 (110.6)^‡^ | 435.5 (89.85)^‡^ |  | 561.4 (596.9) | 585 (363.6) | 796.8 (452.9) |  | 1027 (197)^‡^ | 1040 (166.9)^‡^ | 870.5 (109.9)^‡^ |  | 927.8 (347.6) | 1011 (131.2) | 810.8 (467.4) |
| **sCD23, pg/mL** |  | 1983 (754.3) | 2059 (1217) | 1915 (768.3) |  | 1144 (832.2) | 1269 (867.8) | 1584 (797.8) |  | 1822 (1741) | 1574 (837.8) | 1498 (379.5) |  | 1993 (1805) | 2227 (1474) | 2892 (2694) |
| **κ FLC, mg/L** |  | 13.51 (2.811) | 14.7 (2.754) | 17.02 (6.348) |  | 12.16 (3.315)^‡^ | 11.7 (2.766)^‡^ | 13.37 (1.81)^‡^ |  | 9.845 (2.384) | 11.18 (3.64) | 10.61 (2.248) |  | 14.54 (3.344)^‡^ | 15.08 (4.094)^‡^ | 14.92 (3.247)^‡^ |
| **λ FLC, mg/L** |  | 11.85 (3.098) | 12.92 (6.619) | 13.43 (6.794) |  | 10.04 (2.746) | 10.89 (2.527) | 11.76 (3.497) |  | 7.26 (3.924) | 8.918 (2.272) | 8.89 (3.258) |  | 10.92 (2.906)^‡^ | 10.75 (3.124)^‡^ | 12 (3.596)^‡^ |
| **κ/λ FLC ratio** |  | 1.178 (0.2645)^‡^ | 1.205 (0.3667)^‡^ | 1.125 (0.2889)^‡^ |  | 1.111 (0.4)^‡^ | 1.088 (0.144)^‡^ | 1.118 (0.4122)^‡^ |  | 1.164 (0.3135)^‡^ | 1.206 (0.2308)^‡^ | 0.998 (0.3097)^‡^ |  | 1.282 (0.4605) | 1.435 (0.3905) | 1.284 (0.1473) |
| **IgA, g/L** |  | 1.487 (0.3962) | 2.278 (0.7698) | 2.412 (1.087) |  | 1.595 (0.6216)^‡^ | 1.485 (0.4731)^‡^ | 1.38 (0.6421)^‡^ |  | 1.212 (0.4818)^‡^ | 1.008 (0.4438)^‡^ | 1.356 (0.5186)^‡^ |  | 1.727 (0.5952)^‡^ | 1.362 (0.7211)^‡^ | 1.821 (0.8267)^‡^ |
| **IgG, g/L** |  | 10.42 (0.5052)^‡^ | 11.78 (2.916)^‡^ | 12.78 (4.453)^‡^ |  | 7.421 (2.178) | 8.023 (1.905) | 7.212 (2.042) |  | 5.391 (2.367)^‡^ | 6.063 (2.101)^‡^ | 6.504 (1.878)^‡^ |  | 9.378 (3.492) | 9.876 (1.723) | 9.829 (1.789) |
| **IgM, g/L** |  | 1.526 (0.446) | 1.238 (0.4435) | 1.23 (0.539) |  | 1.005 (0.651) | 1.095 (0.8075) | 1.45 (1.098) |  | 0.829 (1.146) | 0.723 (0.367) | 1.082 (0.7205) |  | 1.027 (1.355) | 0.895 (0.7025) | 1.568 (1.018) |

Abbreviations: BAFF, B cell activating factor; FLC, free light chain; Ig, immunoglobulin; sCD23, soluble CD23.

All significant differences are shown in bold. All p-values were corrected to obtain an adjusted p-value using the method from Benjamini Y, Yekutieli D: The control of the false discovery rate in multiple testing under dependency. Ann Statist 2001:1165-1188.

^†^ Values are expressed as median (interquartile range) unless otherwise indicated.

^‡^ Mean (standard deviation).

* P-value < 0.05; ** P-value < 0.01; *** P-value < 0.001.

**Supplemental Table S2.**

Levels of serum markers of B cell activation in the group of pregnant women by mode of delivery.

|  |  | **Pregnant women (n = 43)^†^** | | | | | | | | | | |
| --- | --- | --- | --- | --- | --- | --- | --- | --- | --- | --- | --- | --- |
|  |  | **3^rd^ trimester** | |  |  | **Day of delivery** | |  |  | **Postpartum period** | |  |
| **Serum marker** |  | Vaginal | Cesarean | P-value‡ |  | Vaginal | Cesarean | P-value^‡^ |  | Vaginal | Cesarean | P-value^‡^ |
| **BAFF, pg/mL** |  | 724.1 (336.2) | 895.2 (771.2) | 1.000 |  | 1079 (305)^§^ | 870.4 (225.9)^§^ | 1.000 |  | 1321 (1744) | 1218 (936) | 1.000 |
| **sCD23, pg/mL** |  | 1988 (1295) | 1673 (1086) | 1.000 |  | 2146 (1284) | 1544 (380) | 1.000 |  | 2548 (1983) | 2628 (1269) | 1.000 |
| **κ FLC, mg/L** |  | 13.14 (5.54)^§^ | 12.57 (2.96)^§^ | 1.000 |  | 10.48 (4.17)^§^ | 10.93 (2.63)^§^ | 1.000 |  | 12.66 (4.24)^§^ | 16.57 (3.63)^§^ | 0.142 |
| **λ FLC, mg/L** |  | 13.21 (8.58) | 11.65 (3.62) | 1.000 |  | 9.54 (3.32) | 10.55 (5.24) | 1.000 |  | 10.1 (4.64)^§^ | 11.58 (2.89)^§^ | 0.591 |
| **κ/λ FLC ratio** |  | 1.05 (0.25)^§^ | 1.10 (0.29)^§^ | 1.000 |  | 1.19 (0.36) | 1.18 (0.36) | 1.000 |  | 1.37 (0.46) | 1.45 (0.29) | 0.591 |
| **IgA, g/L** |  | 1.55 (0.77)^§^ | 1.42 (0.66)^§^ | 1.000 |  | 1.17 (0.38)^§^ | 1.20 (0.82)^§^ | 1.000 |  | 1.49 (0.72)^§^ | 1.8 (1.4)^§^ | 1.000 |
| **IgG, g/L** |  | 8.51 (2.67) | 7.97 (1.88) | 1.000 |  | 6.69 (2.65)^§^ | 6.66 (1.75)^§^ | 1.000 |  | 9.98 (2.4) | 11 (2.91) | 0.591 |
| **IgM, g/L** |  | 1.07 (0.45) | 1.99 (1.88) | 1.000 |  | 1.07 (0.63) | 1.32 (0.93) | 1.000 |  | 1.24 (0.64) | 1.59 (0.81) | 0.855 |

Abbreviations: BAFF, B cell activating factor; FLC, free light chain; Ig, immunoglobulin; sCD23, soluble CD23.

^†^ Values are expressed as median (interquartile range) unless otherwise indicated.

^‡^ Significant differences are shown in bold. All p-values were corrected to obtain an adjusted p-value using the method from Benjamini Y, Yekutieli D: The control of the false discovery rate in multiple testing under dependency. Ann Statist 2001:1165-1188.

^§^ Mean (standard deviation).

**Supplemental Table S3.**

Summary of Spearman’s correlation coefficients and P-values for absolute and relative levels of peripheral blood B cells and serum BAFF in nonpregnant and pregnant women.

| **B cells** |  |  |  |  | **Pregnant women (n = 43)** | | | | | | | |
| --- | --- | --- | --- | --- | --- | --- | --- | --- | --- | --- | --- | --- |
|  |  | **Nonpregnant women (n = 35)** | |  | **3^rd^ trimester** | |  | **Day of delivery** | |  | **Postpartum period** | |
|  |  | *ρ* | P-value^†^ |  | *ρ* | P-value^†^ |  | *ρ* | P-value^†^ |  | *ρ* | P-value^†^ |
| **CD19^+^, cells/μL** |  | 0.0143 | 1.000 |  | -0.0779 | 1.000 |  | -0.0749 | 1.000 |  | -0.0798 | 1.000 |
| **CD19^+^, %** |  | -0.0203 | 1.000 |  | 0.0558 | 1.000 |  | 0.0816 | 1.000 |  | -0.1692 | 1.000 |
| **IgD^+^CD27^-^, cells/μL** |  | 0.1181 | 1.000 |  | -0.0679 | 1.000 |  | -0.0006 | 1.000 |  | -0.0433 | 1.000 |
| **IgD^+^CD27^-^, %** |  | 0.1445 | 1.000 |  | 0.1303 | 1.000 |  | 0.0009 | 1.000 |  | 0.1121 | 1.000 |
| **CD27^+^, cells/μL** |  | -0.0394 | 1.000 |  | -0.1143 | 1.000 |  | -0.0999 | 1.000 |  | -0.2127 | 1.000 |
| **CD27^+^, %** |  | -0.1232 | 1.000 |  | -0.0776 | 1.000 |  | -0.0043 | 1.000 |  | -0.1045 | 1.000 |
| **CD27^+^IgD^+^/MZL, cells/μL** |  | 0.0617 | 1.000 |  | -0.0556 | 1.000 |  | -0.1101 | 1.000 |  | -0.0619 | 1.000 |
| **CD27^+^IgD^+^/MZL, %** |  | -0.0134 | 1.000 |  | 0.0274 | 1.000 |  | -0.0789 | 1.000 |  | 0.0299 | 1.000 |
| **CD27^+^IgD^-^ (with switch), cells/μL** |  | -0.1749 | 1.000 |  | -0.2333 | 1.000 |  | -0.0841 | 1.000 |  | -0.2676 | 1.000 |
| **CD27^+^IgD^-^ (with switch), %** |  | -0.2697 | 1.000 |  | -0.1976 | 1.000 |  | -0.0353 | 1.000 |  | -0.1541 | 1.000 |
| **CD38^++^IgM^hi^, cells/μL** |  | 0.2319 | 1.000 |  | -0.2162 | 1.000 |  | -0.0177 | 1.000 |  | -0.1941 | 1.000 |
| **CD38^++^IgM^hi^, %** |  | 0.3081 | 1.000 |  | -0.1843 | 1.000 |  | 0.0615 | 1.000 |  | -0.2073 | 1.000 |
| **CD19^+^IL10^+^, cells/μL** |  | -0.0331 | 1.000 |  | 0.2502 | 1.000 |  | 0.0685 | 1.000 |  | -0.3040 | 1.000 |
| **CD19^+^IL10^+^, %** |  | -0.1475 | 1.000 |  | 0.2034 | 1.000 |  | 0.1789 | 1.000 |  | -0.2208 | 1.000 |
| **CD19^+^CD24^hi^CD27^+^, cells/μL** |  | 0.0107 | 1.000 |  | 0.1197 | 1.000 |  | 0.0883 | 1.000 |  | -0.1664 | 1.000 |
| **CD19^+^CD24^hi^CD27^+^, %** |  | 0.1094 | 1.000 |  | 0.1629 | 1.000 |  | 0.1456 | 1.000 |  | -0.1564 | 1.000 |
| **CD19^+^CD24^hi^CD38^hi^, cells/μL** |  | 0.2592 | 1.000 |  | 0.0917 | 1.000 |  | -0.0443 | 1.000 |  | -0.1630 | 1.000 |
| **CD19^+^CD24^hi^CD38^hi^, %** |  | 0.2811 | 1.000 |  | 0.1268 | 1.000 |  | 0.0465 | 1.000 |  | -0.1423 | 1.000 |

Abbreviations: BAFF, B cell activating factor; Ig, immunoglobulin; sCD23, soluble CD23.

^†^ Significant correlations are shown in bold. All p-values were corrected to obtain an adjusted p-value using the method form Benjamini Y, Yekutieli D: The control of the false discovery rate in multiple testing under dependency. Ann Statist 2001:1165-1188.
